# Evaluation of edonerpic maleate as a CRMP2 inhibitor for pain relief

**DOI:** 10.1080/19336950.2019.1684608

**Published:** 2019-11-02

**Authors:** Aubin Moutal, Zhiming Shan, Victor G. Miranda, Liberty François-Moutal, Cynthia L. Madura, May Khanna, Rajesh Khanna

**Affiliations:** a Department of Pharmacology, College of Medicine, The University of Arizona Health Sciences, Tucson, AZ, USA; b Department of Anesthesiology, Shenzhen People’s Hospital & Second Clinical Medical College of Jinan University, Shenzhen, P.R. China; c Department of Anesthesiology, The First Affiliated Hospital, Sun Yat-sen University, Guangzhou, P.R. China; d Center for Innovation in Brain Science, University of Arizona, Tucson, AZ, United States

**Keywords:** CRMP2, edonerpic maleate, DRG sensory neuron, ion channels, post-surgical pain

## Abstract

We have previously reported that the microtubule-associated collapsin response mediator protein 2 (CRMP2) is necessary for the expression of chronic pain. CRMP2 achieves this control of nociceptive signaling by virtue of its ability to regulate voltage-gated calcium and sodium channels. To date, however, no drugs exist that target CRMP2. Recently, the small molecule edonerpic maleate (1 -{3-[2-(1-benzothiophen-5-yl)ethoxy]propyl}azetidin-3-ol maleate), a candidate therapeutic for Alzheimer’s disease was reported to be a novel CRMP2 binding compound with the potential to decrease its phosphorylation level in cortical tissues in vivo. Here we sought to determine the mechanism of action of edonerpic maleate and test its possible effect in a rodent model of chronic pain. We observed: (i) no binding between human CRMP2 and edonerpic maleate; (ii) edonerpic maleate had no effect on CRMP2 expression and phosphorylation in dorsal root ganglion (DRG) neurons; (iii) edonerpic maleate-decreased calcium but increased sodium current density in DRG neurons; and (iv) edonerpic maleate was ineffective in reversing post-surgical allodynia in male and female mice. Thus, while CRMP2 inhibiting compounds remain a viable strategy for developing new mechanism-based pain inhibitors, edonerpic maleate is an unlikely candidate.

When searching for novel non-opioid therapeutic targets for chronic pain treatment, we identified the collapsin response mediator protein 2 (CRMP2) as necessary for mechanical allodynia [1]. CRMP2 is localized pre-synaptically in the dorsal horn of the spinal cord where dorsal root ganglia (DRG) sensory neurons synapse onto second-order neurons [2,3]. At this location, CRMP2 controls the presynaptic levels of both CaV2.2 and NaV1.7, two voltage gated ion channels essential for nociceptive signal transmission [3]. Actions of CRMP2 are tightly regulated by post-translational modifications [1]. In chronic neuropathic pain, we found that CRMP2 phosphorylation by cyclin-dependent kinase 5 (Cdk5) on Serine 522 (S522) was sufficient for mechanical allodynia [2] through the facilitation of CaV2.2 and NaV1.7 function [4–8]. By contrast, CRMP2 phosphorylation by the src tyrosine-protein kinase Fyn on tyrosine 32 (Y32) inhibits NaV1.7 [4]. Thus, CRMP2 phosphorylation could be leveraged for the identification of new therapeutics for pain management. Along these lines, a recent study reported the small molecule edonerpic maleate (T-817MA, 1 -{3-[2-(1-benzothiophen-5-yl)ethoxy]propyl}azetidin-3-ol maleate), a candidate therapeutic for Alzheimer’s disease found to increase neurite outgrowth [9], as a novel CRMP2-binding compound with the potential to decrease its phosphorylation level in cortical tissues in vivo [10]. Here we sought to determine the mechanism of action of edonerpic maleate and its possible effect in a rodent model of chronic pain.

Edonerpic maleate was reported to bind to CRMP2 with a Kd of ~735 µM by isothermal titration calorimetry (ITC) [10]. Given this low-affinity binding, we first set out to confirm this through complementary biophysical approaches. We used saturation transfer difference nuclear magnetic resonance (STD-NMR) -a method for studying transient protein–ligand interactions in solution – to assess a direct binding between this small molecule and CRMP2. We tested three different ratios of CRMP2/edonerpic maleate: no binding was detected between CRMP2 and edonerpic maleate (Figure 1(a)). (S)-lacosamide, an inhibitor of CRMP2 phosphorylation [3,5,7], was used as a positive control at the same concentrations and showed binding to CRMP2 (not shown), consistent with our previously published finding [7]. Next, we used microscale thermophoresis (MST), a method for the biophysical analysis of biomolecular interactions, but were unable to detect any interaction between edonerpic maleate and CRMP2 with this method either (not shown). Thus, our results argue against a direct binding between CRMP2 and this small molecule. The differences between our results and previous findings may reflect differences of sensitivity between these methodologies as the Kd obtained by ITC required utilization of ~10 fold more CRMP2 than our studies. Notwithstanding the incongruency between our findings and those of Abe et al. [10] we pursued further exploration of the biological effect of edonerpic maleate on CRMP2.

**Figure 1.**
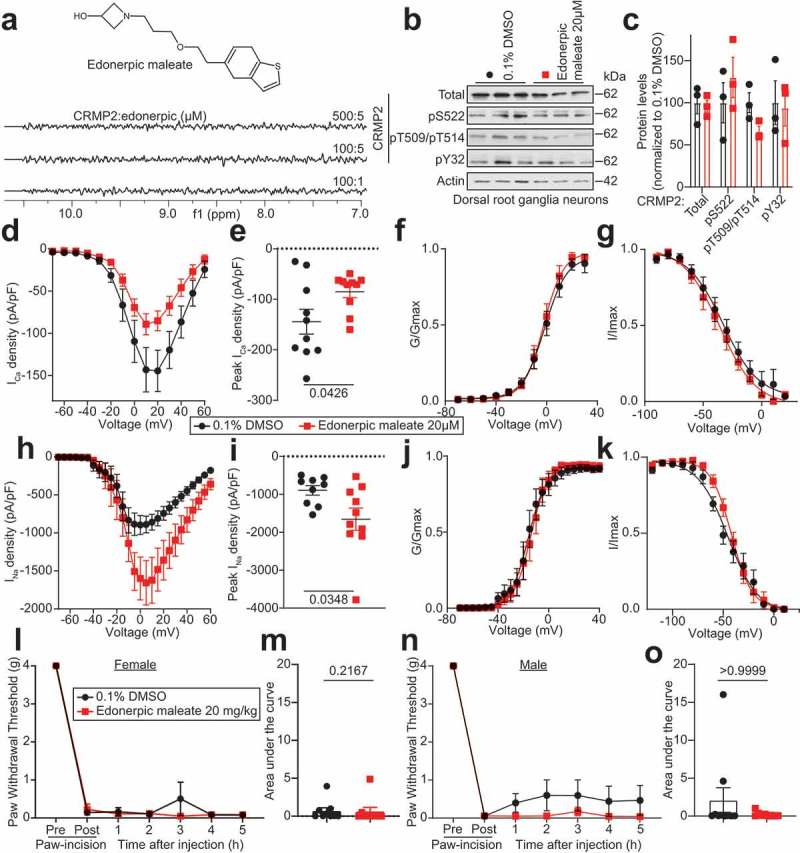
Evaluation of edonerpic maleate as an inhibitor of CRMP2. (a) ^1^D^1^H saturation transfer difference (STD) magnetic nuclear resonance (NMR) STD-NMR showing on-resonance difference spectrum of three different CRMP2:edonerpic ratios (in µM). The aromatic region of the NMR spectrum (5.5–9 ppm) is shown. (b) representative immunoblot of CRMP2 expression and phosphorylation level in DRG neurons in culture treated with edonerpic maleate (20 µM) overnight. (c) Bar graph with scatter plot showing no difference of CRMP2 expression and phosphorylation level after edonerpic treatment (n=3). Summary of the normalized (pA/pF) total calcium current (I_Ca_) density versus voltage relationship (d) and peak total Ca^2+^ current density at +10 mV (mean ± SEM) (E) from DRG sensory neurons treated as indicated (n=10 each). Boltzmann fits for normalized conductance G/G_max_ voltage relations for voltage-dependent activation (f) and inactivation (g) of sensory neurons treated as indicated. Summary of the normalized (pA/pF) total sodium current (I_Na_) density versus voltage relationship (h) and peak total Na^2+^ current density at +10 mV (mean ± SEM) (I) from DRG sensory neurons treated as indicated (n=9 for 0.1%DMSO and n=10 for edonerpic maleate). Boltzmann fits for normalized conductance G/G_max_ voltage relations for voltage-dependent activation (j) and inactivation (k) of sensory neurons treated as indicated. After a paw incision surgery, both male and female mice developed mechanical allodynia at 24 h. Paw withdrawal threshold of adult (L) female (n = 9) or (n) male (n=9) was measured after injection with edonerpic maleate (20 mg/kg, i.p.) or vehicle (0.1% DMSO in saline). Area under the curve was derived for (m) female or (o) male mice. Experimenter was blinded to the treatment condition. Exact p-values are indicated for each panel (Mann–Whitney test).

First, we tested if edonerpic maleate could alter the levels of CRMP2 phosphorylation in cultured DRG sensory neurons. After an overnight incubation with the compound (20 µM), CRMP2 expression levels were unchanged (Figure 1(b–c)). CRMP2 phosphorylation by Cdk5 (S522), glycogen synthase kinase 3 beta (GSK3β; T509/T514, which requires prior Cdk5 phosphorylation of CRMP2 [11]) and by the Fyn (Y32) were not significantly altered by edonerpic maleate (Figure 1(b–c)). Thus, edonerpic maleate is unable to control the CRMP2 phosphorylation level in DRG neurons.

CRMP2 functions, such as regulation of ion channels, may be linked to its ability to form tetramers [12]. Edonerpic maleate was found to disrupt CRMP2 tetramers [10]. Our previous body of work has established a role for phosphorylated and non-phosphorylated CRMP2 in control of voltage-gated calcium and sodium channels [1,3–8,13–22]. Therefore, we tested the effect of edonerpic maleate on these channels. Cultured DRG neurons were incubated with 20 µM of edonerpic maleate overnight and calcium currents recorded (Figure 1(d)). Edonerpic maleate inhibited peak calcium current density (p = 0.0426, Mann–Whitney test, Figure 1(e)) compared to 0.1% DMSO-treated controls neurons but had no effect on activation (Figure 1(f)) or inactivation (Figure 1(g)) properties of these channels. These results show that edonerpic maleate can decrease the function of voltage-gated calcium channels in DRG sensory neurons, in a manner similar to our results obtained by uncoupling CRMP2 from CaV2.2 using decoy peptides [15,21–26]. Next, we tested if edonerpic maleate could inhibit the voltage-gated sodium channels. Cultured DRG neurons were treated overnight with 20 µM of edonerpic maleate prior to sodium current recordings (Figure 1(h)). In these experiments, neurons treated with edonerpic maleate had increased peak sodium current density compared to 0.1% DMSO-treated controls (p = 0.0348, Mann–Whitney test, Figure 1(i)). Activation and inactivation properties of voltage-gated sodium channels were unchanged (Figure 1(j–k)). These results are in diametric opposition to our previous findings which demonstrated maintenance, not augmentation, of sodium channel current density by CRMP2 [4].

Despite the opposing effect on calcium and sodium channels, we next asked whether edonerpic maleate could be beneficial for pain. As calcium channels are known mediators of post-surgical allodynia [5,21], we evaluated the anti-allodynic potential of edonerpic maleate in a mouse model of post-surgical pain [27]. Female and male mice were subjected to a paw incision which resulted in allodynia at 24 h after injury and then treated intraperitoneally with edonerpic maleate (20 mg/kg, i.p.). Edonerpic maleate failed to reverse post-surgical allodynia in the 5-h window following injection (Figure 1(l–o)). These results show that although edonerpic maleate treatment results in inhibition of calcium currents in sensory neurons, it fails to reverse allodynia.

Together, these results show that edonerpic maleate is unlikely to be a CRMP2 targeting small molecule. We base this conclusion on (i) our inability to detect any binding between edonerpic maleate and CRMP2, and (ii) the observation that edonerpic maleate treatment of DRG sensory neurons had no effect on CRMP2 expression and phosphorylation. That edonerpic maleate decreased calcium but increased sodium current density in DRG neurons likely accounts for why the compound was ineffective in reversing post-surgical allodynia. In contrast, subverting CRMP2 phosphorylation with (S)-lacosamide not only decreases calcium currents but is also able to reverse post-surgical allodynia, supporting our premise that CRMP2 inhibiting compounds could be useful for mitigating pain. However, edonerpic maleate does not meet the criteria as a CRMP2 inhibitor. Recently, edonerpic maleate showed no clinical efficacy in patients with mild to moderate Alzheimer’s disease [28]. This clinical study with our pre-clinical results may indicate that the therapeutic actions of Edonerpic maleate could be specific to stroke.

## Methods

### Animals

As done previously [3], adult female Sprague Dawley rats (for electrophysiology experiments, Pathogen-free; 100 g; Envigo) or CD1 mice (19–22 g, Charles River) were housed in light and temperature-controlled conditions (12-h light/12-h dark cycle; lights on 07:00–19:00; 23 ± 3°C), fed *ad libitum* with standard rodent chow and water. All experiments and procedures were conducted in accordance with the regulations of the Institutional Animal Care and Use Committee of the University of Arizona’s College of Medicine and the NIH-published Guide for Care and Use of Laboratory Animals’, as well as the ethical regulations of the International Association for the Study of Pain. With regard to experimental design, behavioral experiments were done with random assignment of animals to both treatment and control conditions; experimenters were blinded to both experimental groups and treatments.

### Saturation transfer difference nuclear magnetic resonance spectroscopy

^1^D^1^H saturation transfer difference nuclear magnetic resonance (STD NMR) spectra with a spectral width of 12 ppm were collected for samples containing 500 or 100 µM endonerpic compound with either 1 or 5 µM CRMP2-His (1:100 dilution was always maintained) in PBS, 10% D_2_O. STD NMR spectra were collected with a spectral width of 12 ppm, 16 K data points, and 3 second repetition delay. A saturation of the protein was achieved by a 2 second train of selective 50 ms Gaussian pulses centered at 0.74 ppm (on-resonance) and 30 ppm (off resonance). A 20-ms spin-lock was used to suppress the protein signal, followed by the double PFG spin echo to remove residual water signal. We acquired 512 scans per experiment. The on-resonance and off-resonance spectra were acquired interleaved, and the difference spectrum was acquired by phase cycling. Spectra processing and analysis were performed with the VNMRJ 3.2 (Agilent Technologies, Santa Clara, CA) and MestReNova 7.1 (Mestrelab Research, S.L., Santiago de Compostela, Spain).

### Immunoblot preparation and analysis

DRG neurons in culture were treated with 20 µM of edonerpic maleate overnight or with vehicle (0.1% DMSO). Lysates were generated by homogenization in RIPA buffer (50 mM Tris-HCl, pH 7.4, 50 mM NaCl, 2 mM MgCl_2_, 1% [vol/vol] NP40, 0.5% [mass/vol] sodium deoxycholate, 0.1% [mass/vol] SDS) as described previously [4]. Protease inhibitors (Cat# B14002; Bimake, Houston, TX), phosphatase inhibitors (Cat# B15002, Bimake), and benzonase (Cat#71206, Millipore, Billerica, MA). Protein concentrations were determined using the BCA protein assay (Cat# PI23225, Thermo Fisher Scientific, Waltham, MA). Indicated samples were loaded on 4–20% Novex® gels (Cat# EC60285BOX, Thermo Fisher Scientific, Waltham, MA). Proteins were transferred for 1 h at 120 V using TGS (25 mM Tris pH = 8.5, 192 mM glycine, 0.1% (mass/vol) SDS), 20% (vol/vol) methanol as transfer buffer to polyvinylidene difluoride (PVDF) membranes 0.45 μm (Cat# IPVH00010, Millipore, Billerica, MA), pre-activated in pure methanol. After transfer, the membranes were blocked at room temperature for 1 h with TBST (50 mM Tris-HCl, pH 7.4, 150 mM NaCl, 0.1% Tween 20), and 5% (mass/vol) nonfat dry milk, then incubated separately with the indicated primary antibodies CRMP2 (Sigma-Aldrich, Cat# C2993), CRMP2 pTyr32 (generously provided by Dr. Yoshio Goshima [5]), CRMP2 pThr509/Thr514 (MRC, Cat# PB-043,), CRMP2 pSer522 (ECM Biosciences, Cat# CP2191) and Actin (Sigma-Aldrich, Cat# A2066) in TBST, and 5% (mass/vol) BSA, overnight at 4°C. Following incubation in horseradish peroxidase-conjugated secondary antibodies from Jackson immune research, blots were revealed by enhanced luminescence (WBKLS0500, Millipore, Billerica, MA) before exposure to photographic film. Films were scanned, digitized, and quantified using Un-Scan-It gel version 7.1 scanning software by Silk Scientific Inc. For all experiments, CRMP2 phosphorylation levels were always normalized to total CRMP2 levels in the same sample.

### Preparation of acutely dissociated dorsal root ganglion neurons

Dorsal root ganglia from all levels were acutely dissociated using methods as described previously [4]. Rat DRG neurons were isolated from 100 g Sprague-Dawley rats using previously developed procedures [9]. In brief, removing dorsal skin and muscle and cutting the vertebral bone processes parallel to the dissection stage-exposed DRG. Dorsal root ganglia were then collected, trimmed at their roots, and enzymatically digested in 3 mL bicarbonate-free, serum-free, sterile DMEM (Cat# 11965, Thermo Fisher Scientific) solution containing neutral protease (3.125 mg.ml^−1^, Cat#LS02104; Worthington, Lakewood, NJ) and collagenase type I (5 mg/mL, Cat# LS004194, Worthington, Lakewood, NJ) and incubated for 60 min at 37°C under gentile agitation. Dissociated DRG neurons (~1.5 × 10^6^) were then gently centrifuged to collect cells and washed with DRG media DMEM containing 1% penicillin/streptomycin sulfate from 10,000 μg/mL stock, 30 ng/mL nerve growth factor, and 10% fetal bovine serum (Hyclone) before plating onto poly-D-lysine – and laminin-coated glass 12- or 15-mm coverslips. Small diameter neurons were selected to target Aδ- and c-fiber nociceptive neurons. For rat DRG cultures, small cells were considered to be ~ < 30 μm. All cultures were used within 48 h.

### Whole-cell patch recordings of Ca^2+^ currents in acutely dissociated dorsal root ganglion neurons

Recordings were obtained from acutely dissociated DRG neurons as described previously [10,11]. To isolate calcium currents, Na^+^ and K^+^ currents were blocked with 500 nM tetrodotoxin (TTX; Alomone Laboratories) and 30 mM tetraethylammonium chloride (TEA-Cl; Sigma). Extracellular recording solution (at ~310 mOsm) consisted of the following (in mM): 110 *N*-methyl-D-glucamine (NMDG), 10 BaCl_2_, 30 TEA-Cl, 10 HEPES, 10 glucose, pH at 7.4, 0.001 TTX, 0.01 nifedipine. The intracellular recording solution (at ~310 mOsm) consisted of the following (in mM): contained 150 CsCl_2_, 10 HEPES, 5 Mg-ATP, 5 BAPTA, pH at 7.4. Activation of I_Ca_ was measured by using a holding voltage of −90 mV with voltage steps 200 ms in duration applied at 5-s intervals in +10 mV increments from −70 to +60 mV. Current density was calculated as peak I_Ca_/cell capacitance. Steady-state inactivation of I_Ca_ was determined by applying an 800-ms conditioning prepulse (−100 to −20 mV in +10 mV increments) after which the voltage was stepped to −20 mV for 200 ms; a 15-s interval separated each acquisition.

Whole-cell voltage clamp recordings were performed at room temperature (RT) using an EPC 10 Amplifier-HEKA as previously described [6]. The internal solution for voltage clamp sodium current recordings contained (in millimolar): 140 CsF, 1.1 CsEGTA, 10 NaCl, and 15 HEPES (pH 7.3, 290–310 mOsm/L) and external solution contained (in millimolar): 140 NaCl, 3 KCl, 30 tetraethylammonium chloride, 1 CaCl2, 0.5 CdCl2, 1 MgCl2, 10 D-glucose, and 10 HEPES (pH 7.3, 310–315 mosM/L).

DRG neurons were subjected to current-density (I-V) and fast-inactivation voltage protocols as previously described [4,12]. In the I-V protocol, cells were held at a −80 mV holding potential before depolarization by 20-ms voltage steps from −70 to +60 mV in 5-mV increments. This allowed for collection of current density data to analyze activation of sodium channels as a function of current vs voltage and also peak current density, which was typically observed near ~0 to 10 mV and normalized to cell capacitance (pF). In the fast-inactivation protocol, cells were held at a − 80 mV holding potential prior to hyperpolarizing and repolarizing pulses for 500 ms between −120 and −10 mV in 5 mV increments. This step conditioned various percentages of channels into fast-inactivated states so that a 0-mV test pulse for 20 ms could reveal relative fast inactivation normalized to maximum current. Fire-polished recording pipettes, 2 to 5 MΩ resistance were used for all recordings. Whole-cell recordings were obtained with a HEKA EPC-10 USB (HEKA Instruments Inc., Bellmore, NY); data were acquired with a Patchmaster (HEKA) and analyzed with a Fitmaster (HEKA). Capacitive artifacts were fully compensated, and series resistance was compensated by ~70%. Recordings made from cells with greater than a 5 mV shift in series resistance compensation error were excluded from analysis. All experiments were performed at room temperature (~23°C).

The Boltzmann relation was used to determine the voltage dependence for activation of I_Ca_ and I_Na_ wherein the conductance–voltage curve was fit by the equation G/G_max_ = 1/[1 + exp (V_0.5_ − V_m_)/k], where G is the conductance G = I/(V_m_−E_Ca_ or E_Na_), G_max_ is the maximal conductance obtained from the Boltzmann fit under control conditions, V_0.5_ is the voltage for half-maximal activation, V_m_ is the membrane potential, and k is a slope factor. E_Ca_ is the reversal potential for I_Ca_; E_Na_ is the reversal potential for I_Na_ and was determined for each individual neuron. The values of I_Ca_ and I_Na_ around the reversal potential were fit with a linear regression line to establish the voltage at which the current was zero. The Boltzmann parameters were determined for each individual neuron and then used to calculate the mean ± S.E.M.

## Surgeries and behavioral analysis

Mouse paw incision and pSNL surgeries were done following published methods [13]. Allodynia was tested as described previously [3,14]. Data was analyzed as described by Chaplan et al. [15] using the nonparametric method of Dixon.

## Statistics

Statistical analyses were performed using GraphPad Prism 8 (GraphPad, La Jolla, CA). Data were sourced from a minimum of three independent biological replicates unless indicated otherwise. All data represent the mean ± S.E.M. The statistical significance of differences between groups was determined by non-parametric Student’s t-test, analysis of variance (ANOVA) followed by post hoc comparisons (Tukey) using Prism 8. Statistical significance was set at p < 0.05.
